# Markers of Epithelial–Mesenchymal Transition and Mucinous Histology Are Significant Predictors of Disease Severity and Tumor Characteristics in Early-Onset Colorectal Cancer

**DOI:** 10.3390/diagnostics14141512

**Published:** 2024-07-13

**Authors:** Aleksandra Djikic Rom, Sandra Dragicevic, Radmila Jankovic, Sanja Radojevic Skodric, Predrag Sabljak, Velimir Markovic, Jovana Rosic Stojkovic, Goran Barisic, Aleksandra Nikolic

**Affiliations:** 1 Department of Pathology, Pathohistology and Medical Cytology, University Clinical Center of Serbia, Pasterova 2, 11000 Belgrade, Serbia; 2 Gene Regulation in Cancer Group, Institute of Molecular Genetics and Genetic Engineering, University of Belgrade, 11000 Belgrade, Serbia; sandra.d@imgge.bg.ac.rs (S.D.); aleksni@imgge.bg.ac.rs (A.N.); 3 Institute of Pathology, Faculty of Medicine, University of Belgrade, 11000 Belgrade, Serbia; radmila.jankovic011@gmail.com (R.J.); sanjaskodric@gmail.com (S.R.S.); 4 Faculty of Medicine, University of Belgrade, 11000 Belgrade, Serbia; predrag.sabljak@med.bg.ac.rs (P.S.); velimir.markovic@med.bg.ac.rs (V.M.); jovana.rosic@med.bg.ac.rs (J.R.S.); goran.barisic@med.bg.ac.rs (G.B.); 5 Clinic for Digestive Surgery—First Surgical Clinic, University Clinical Center of Serbia, 11000 Belgrade, Serbia

**Keywords:** early-onset colorectal cancer, epithelial–mesenchymal transition, mucinous tumor

## Abstract

Approximately 20% of patients with colorectal cancer (CRC) are diagnosed with a mucinous subtype of this tumor, have a worse prognosis, and often show resistance to available therapies. Molecules from the mucin family are involved in the regulation of epithelial–mesenchymal transition (EMT), which significantly determines the cancer aggressiveness. This study aimed to examine the diagnostic and prognostic significance of mucinous histology and EMT markers in patients with early-onset CRC and their association with disease severity and tumor characteristics. This study included tumor tissue samples from 106 patients diagnosed with CRC before the age of 45, 53 with mucinous and 53 with non-mucinous tumors. The EMT status was determined by immunohistochemical analysis of E-cadherin and Vimentin in tissue sections. Mucinous tumors had significantly higher Mucin-1 (*p* < 0.001) and cytoplasmic E-cadherin (*p* = 0.043) scores; they were significantly less differentiated (*p* = 0.007), more advanced (*p* = 0.027), and predominately affected right the colon (*p* = 0.039) compared to non-mucinous tumors. Epithelial tumors were significantly better differentiated (*p* = 0.034) and with less prominent tumor budding (*p* < 0.001) than mesenchymal tumors. Mucin-1 and Vimentin were independent predictors of tumor differentiation (*p* = 0.006) and budding (*p* = 0.001), respectively. Mucinous histology and EMT markers are significant predictors of disease severity and tumor characteristics in early-onset colorectal cancer.

## 1. Introduction

Colorectal cancer (CRC) is the third most commonly diagnosed cancer in the world, including both sexes, and the second most common cause of cancer death according to the GLOBOCAN data [1]. The number of patients with this cancer is constantly increasing, especially in the population younger than 50 years [2]. At the time of diagnosis, the median age of patients with colon cancer is 68 years in men and 72 years in women, and the median age in patients with rectal cancer is 63 years for both sexes. According to the Surveillance, Epidemiology and End Results Program (SEER) database in the United States, about 5% of all cases of CRC are diagnosed in patients under 45 years of age [2]. A concerning increase in CRC incidence among younger individuals has resulted in altered recommendations for screening, and contemporary guidelines indicate 45 years as the age cutoff for early-onset disease [3,4,5].

Sporadic early-onset CRC is much more common than the hereditary subtype, and recent reports suggest that its molecular properties are different from late-onset tumors [6,7]. Early-onset disease is more often characterized by aggressive tumor histology, more distal tumor localization (descending, sigmoid colon, and rectum), mucinous histology or signet ring cell histology, advanced and metastatic stage of the disease, higher tumor grade, higher rate of perineural invasion, and a positive resection margin [8,9,10].

Mucinous histology of the tumor observed in 10–20% of CRC patients is characterized by an abundance of extracellular mucin component that accounts for at least 50% of the tumor volume [11,12]. In terms of clinical pathology, this CRC subtype is significantly more common in the proximal colon than in the rectum or distal colon, shows an increased lymph node infiltration and peritoneal implants, is larger at the time of diagnosis, and is more commonly diagnosed in younger women [11]. It is most often diagnosed at an advanced stage, has worse prognosis, and often shows resistance to available therapies, subsequently leading to lower overall survival compared to non-mucinous CRC [11,12]. However, the prognostic value of mucinous histology still remains uncertain.

The process of epithelial–mesenchymal transition (EMT) is essential for embryonal development, but it also occurs during the development of epithelial tumors, causing cellular transmigration that eventually leads to metastasis. E-cadherin and Vimentin are widely regarded as key markers for EMT [13,14]. E-cadherin, typically expressed in epithelial cells, is a hallmark of the epithelial phenotype, and its loss is associated with EMT [14]. On the other hand, Vimentin is a mesenchymal marker, and its expression is indicative of the mesenchymal phenotype, which cells acquire during EMT [14,15]. In general, EMT is characterized by the decrease in the function of epithelial markers such as E-cadherin, which plays an important role in epithelial cell adhesion and tissue architecture, and an increase in the expression of mesenchymal markers such as Vimentin, which is important for maintaining cell shape and integrity [13]. Loss of E-cadherin leads to a loss of epithelial differentiation and the acquisition of motility and an invasiveness phenotype, and its loss is associated with progression and poor prognosis of CRC, tumor invasiveness, metastases, and increased resistance to apoptosis. The EMT process is mediated and orchestrated by various transcription modulators and a number of signaling intermediates, including mucins that play a significant role in the process of cell differentiation and are associated with the aggressive behavior of metastatic tumor cells [16,17].

The members of the mucin family of glycoproteins are secreted by epithelial cells and form extracellular mucinous gel in human tissues. Mucin-1 is aberrantly overexpressed in CRC, participates in the regulation of the metabolic program, activates antiapoptotic proteins, and induces drug resistance. These observations identify Mucin-1 as an attractive marker for CRC diagnosis, immunotherapy, and prognosis. Since CRC patients with high Mucin-1 expression in tumor tissue have a higher risk of metastasis, the level of Mucin-1 expression is important in guiding treatment plans, so determining Mucin-1 expression in CRC by immunohistochemical methods may be important for determining treatment strategies in a clinical setting [17,18]. Mucin-1 can induce the expression of multiple growth factors in the survival and proliferation of tumor cells and the production of angiogenesis factors that promote the formation of new blood vessels in tumor tissues. Overexpression of Mucin-1 was associated with EMT and cell invasion [13,16,18].

The aim of this study was to examine the diagnostic and prognostic significance of mucinous histology and epithelial–mesenchymal transition (EMT) markers in patients with early-onset CRC, as well as their association with disease severity and tumor characteristics.

## 2. Materials and Methods

### 2.1. Study Subjects

This retrospective study included 106 patients who underwent surgical resection of the primary CRC at the Clinic for Digestive Surgery, University Clinical Center of Serbia, in the period from 2006 to 2020. Patients aged 18–45 years were included in the study and divided into two groups according to tumor types, determined based on extracellular mucin production: 53 patients with mucinous adenocarcinoma (mucinous component comprises more than 50% of tumor volume) and 53 patients with non-mucinous adenocarcinoma (no mucinous component in the tumor). Patients with partial mucin production (below 50%) were excluded from the study. Exclusion criteria were the presence of any type of hereditary polypoid or non-polypoid syndrome, chronic inflammatory bowel disease (ulcerative colitis and Crohn’s disease), any type of neoadjuvant chemoradiotherapy, and the presence of other histological, both primary and metastatic, types of carcinomas. This study was conducted according to the guidelines of the Declaration of Helsinki and approved by the Ethical Committee of the University Clinical Center of Serbia (Ref. No.: 175/1, Date: 27 April 2021). Informed consent was obtained from all study participants.

The following data were collected for all subjects: age, sex, tumor localization, degree of tumor differentiation, percentage of extracellular mucin tumor production, tumor classification (using histopathological criteria based on the fifth edition of the World Health Organization (WHO) classification), and tumor disease stage using criteria of the TNM classification of the eighth edition of the American Joint Committee on Cancer from 2017 [19]. Stages of tumor disease were also determined using Dukes and Astler-Coller systems [20,21]. Also, data on lymphovascular and perineural invasion, as well as tumor residual status, were collected for all patients.

### 2.2. Immunohistochemical Analysis

The immunohistochemical (IHC) analysis of E-cadherin, Vimentin, Mucin-1, and pancytokeratin was performed on five tissue sections from a single paraffin block for each subject included in the study. Pancytokeratin was used for better visualization of cancer cells and more precise determination of the tumor budding degree. Tissue sections for IHC staining were cut successively to a thickness of 4 µm on Superfrost Plus plates (Thermo Scientific, Pittsburgh, PA, USA). The process of deparaffinization and rehydration of tissue sections was performed in accordance with standard procedure [22]. Pretreatment for vimentin and pancytokeratin was performed in Target Retrieval Solution, High Ph (50×), while for E-cadherin and Mucin-1, it was performed in Target Retrieval Solution, Low Ph (50×), brand EnVision FLEX in PTLink (Dako, Glostrup, Denmark). After pretreatment, automatic staining was performed for Vimentin (monoclonal mouse antibody NCL-L-VIMENTIN; dilution 1:400) and pancytokeratin (monoclonal mouse antibody AE1/AE3; DAKO; dilution 1:100) in AUTOSTAINER LINK48 (Dako, Glostrup, Denmark), while for Mucin-1 (monoclonal mouse antibody MRQ-17, Cell Marque; dilution 1:100), automatic staining was performed in AUTOSTEINER 360 (Thermo Scientific). Automatic staining was performed in accordance with the manufacturer’s instructions. After staining, contrast staining was performed on all sections with the help of Mayer’s hematoxylin. The detection system for vimentin and pancytokeratin was performed using EnVision FLEX, and for Mucin-1 using Mouse/Rabbit PolyDetector Plus; Bio SB. After pretreatment, staining with E-cadherin was performed manually using a monoclonal mouse antibody (SPM471; SANTA CRUZ; sc56527; dilution 1:50). The detection was performed using Mouse/Rabbit PolyDetector Plus; Bio SB. Evaluation of IHC staining was performed using a standard Leica DM1000 light microscope. After selection, the representative fields were photographed using a Leica ICC50E camera, and the QuPath free software, version 0.2.3. (QuPath developers, The University of Edinburgh, Scotland) was used for counting. The status of the EMT process was determined based on the combined analysis of E-cadherin and Vimentin expression. Tumors positive for E-cadherin and/or negative for Vimentin were considered epithelial, while tumors negative for E-cadherin and/or positive for Vimentin were classified as mesenchymal.

### 2.3. Evaluation of E-Cadherin Expression

For evaluation of E-cadherin expression, the entire cross-section at ×4 and ×10 lens magnification was first examined to find the infiltrative zone of tumor spread that was the only one to be assessed at ×40 lens magnification by observing at least 100 cancer cells. Both membrane and cytoplasmic staining were evaluated. For the cell membrane, a four-level scale was used: (1) +++ for continuous staining of the membrane with the creation of a honeycomb-shaped pattern; (2) ++ for continuous staining present in 40–90% of membranes; (3) + for continuous staining present in 10–39% of membranes; (4)—for staining in <10% membranes. Cytoplasmic staining was also classified into four categories: (1) 0 for no noticeable staining; (2) 1 for weak but still noticeable staining; (3) 2 for moderate, obviously positive, but still weak staining; (4) 3 for strong, intense staining. The membrane (MI) and cytoplasmis (CI) staining indexes were calculated based on the intensity of membrane or cytoplasmic staining, and the proportion of positively stained cancer cells taken into account, using the following formula, I = 0 × f0 + 1 × f1 + 2 × f2 + 3 × f3, where I is the staining index, with f0–f3 cell fractions showing a defined level of staining intensity (0 to 3). Theoretically, the staining index is in a range between 0 and 3 [23,24]. Values of staining index greater than 0.5 were considered positive.

### 2.4. Evaluation of Vimentin Expression

Vimentin expression in cancer cells was first assessed by examining the cross-section as a whole under low-power magnification (lens ×4) and then confirmed by high-power magnification (lens ×20 and ×40). At least ten visual fields were observed, predominantly in the region of the tumor-invasive front, in parallel with the same visual fields used for E-cadherin staining. The immunoreactivity scoring system was applied according to the following two criteria: (1) the proportion of positively stained cells: 0 for 0%, 1 for ≤1%, 2 for 1–10%, 3 for 11–33%, 4 for 34–66% and 5 for 67–100%; and (2) color intensity: 0 for colorless, 1 for pale, 2 for yellow, 3 for brown. The overall Vimentin score was calculated according to the modified Allerd scoring system by summing the two criteria into a single score: 0–1 negative, 2–3 weak positivity, 4–6 moderate positivity, 7–8 strong positivity [13,25].

### 2.5. Evaluation of Mucin-1 Expression

For evaluation of Mucin-1 expression, the selected sections were first examined in full at ×4 and ×10 lens magnification, and since very heterogeneous antibody expression occurs at individual sections, 10 HPF visual fields were used, and counting was performed using ×40 lens magnification [26]. The immunostaining intensity of individual cells was evaluated on a scale from 0 (no staining) to +4 (strongest intensity). In addition, the percentage of stained cells for each of the intensities was determined. The percentage of cells at each intensity was multiplied by the appropriate intensity value to obtain an immunohistochemical score ranging from 0 to 4. The value of the score ≥0.5 or at least 25% of tumor cells was considered positive expression [27]. For the samples with positive expression, the degree of positivity was determined: 0 for no positive cells, 1 for less than 5%, 2 for 5–29%, 3 for 30–59%, and 4 for more than 60% of positive cells [28].

### 2.6. Pancytokeratin Staining and Tumor Budding Evaluation

Pancytokeratin immunostaining was performed to facilitate identification of cancer cells and tumor budding evaluation. Pancytokeratin staining was scored as follows: 0 for no staining; 1 for less than 5% of tumor cells; 2 for 5–25% of tumor cells; 3 for 25–50% of tumor cells; 4 for more than 50% of tumor cells [29]. To determine the degree of tumor budding, the H&E-stained section with the largest degree of buds was selected; then, a hot spot was identified on the ×10 lens. The buds were counted in the selected hot spot at ×20 lens, and tumor budding was scored using a three-step system proposed by the International Consensus Conference on Tumor Budding (ITBCC), as used by the Japan Colon Cancer Association and Rectum: 0–4 buds—low tumor budding (Bd1); 5–9 buds—moderate tumor budding (Bd2); 10 or more buds—high tumor budding (Bd3) [30].

### 2.7. Statistical Analysis

Statistical analyses were performed using Statistical Package for Social Sciences 21.0 (SPSS Inc., Chicago, IL, USA). Data were expressed as mean ± standard deviation (SD) for continuous variables and percentages for categorical variables. The normality of continuous data and homogeneity of variance were tested by one-sample Kolmogorov–Smirnov test and Levene’s test, respectively. Differences between groups for categorical data were analyzed by Fisher’s Exact test and Pearson’s Chi-squared test, while for continuous data, Independent Samples Mann–Whitney U test was used. Logistic regression was performed to analyze the impact of each independent variable on the likelihood of an event of interest. Curves of probabilities for overall survival (OS) were constructed using the Kaplan–Meier product-limit method; the median of survival analysis with a corresponding 95% confidence interval (CI) was used for description, and the log-rank test was utilized for testing differences between curves. *p* value less than 0.05 was considered statistically significant.

## 3. Results

**Figure 1:**
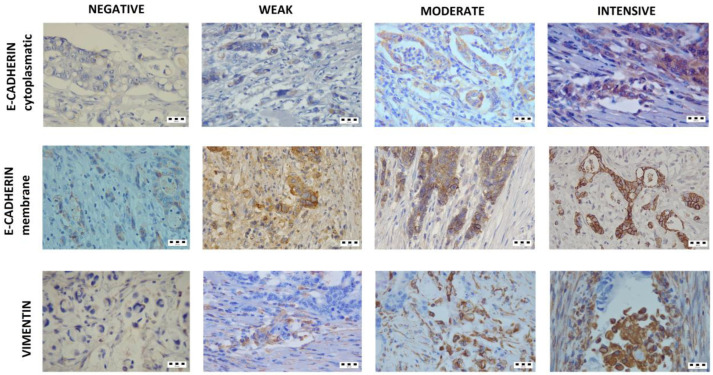
Immunohistochemical staining for epithelial–mesenchymal markers (magnification ×40). Intensity of E-cadherin cytoplasmatic and Vimentin staining (from left to right): colorless scored 0; pallide-flavens scored 1; yellow scored 2; and brown scored 3. Intensity of E-cadherin membrane staining (from left to right): intensive for continuous staining of the membrane with the creation of a honeycomb-shaped pattern; moderate for continuous staining present in 40–90% of membranes; weak for continuous staining present in 10–39% of membranes; negativefor staining in <10% membranes.

The expressions of epithelial marker E-cadherin and mesenchymal marker Vimentin were analyzed in tumor tissue of 106 patients with colorectal cancer diagnosed before the age of 45 years. The patients were recruited for two groups according to the tumor histology—mucinous (53 patients) and non-mucinous (53 patients). The tissue sections were selected to contain the invasive tumor front, and the E-cadherin and Vimentin score were determined (Figure 1). In addition to the total E-cadherin score, membrane and cytoplasmic scores were also analyzed. The clinical and pathological characteristics of patients and scores for analyzed EMT markers are shown in Table 1.

Mucinous tumors were significantly less differentiated than non-mucinous tumors (*p* = 0.007), and they were characterized by a significantly more advanced stage (*p* = 0.027). Also, mucinous tumors affected the right colon significantly more frequently than other tumor locations (*p* = 0.039). Lower average expression of E-cadherin and higher average expression of Vimentin were detected in non-mucinous vs. mucinous tumors. In comparison to non-mucinous, mucinous tumors had a significantly higher cytoplasmic E-cadherin score (*p* = 0.043). Total and membrane E-cadherin scores were also higher in patients with mucinous tumors, although without statistical significance. More non-mucinous tumors were vimentin-positive tumors than mucinous (22.6% vs.16.9%), and the average Vimentin score was higher in non-mucinous vs. mucinous tumors (0.85 ± 1.73 vs. 0.62 ± 1.47).

**Figure 2:**
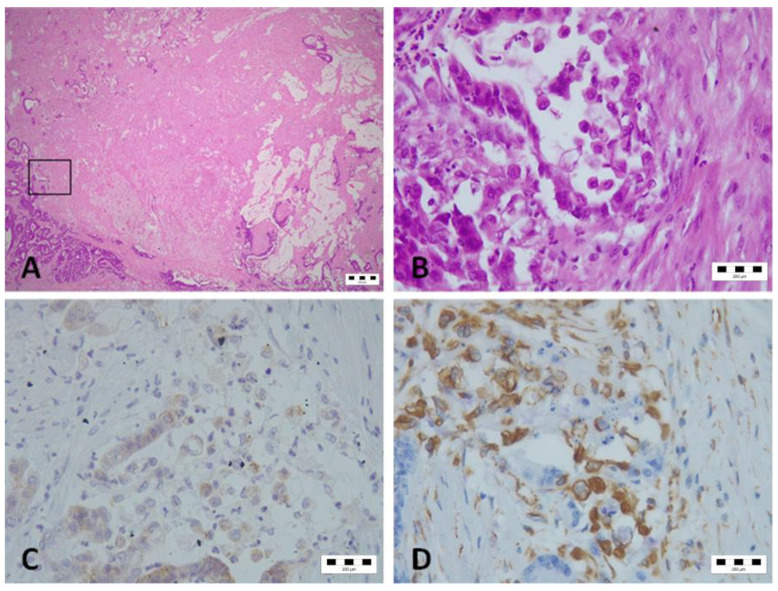
Representative mesenchymal tumor within the group of mucinous tumors. (**A**) The black square highlights the invasive front where EMT was observed (H&E, magnification ×2.5); (**B**) detailed view of the invasive front (H&E, magnification ×40); (**C**) very pale and negative immunohistochemical staining for E-Cadherin (magnification ×40); (**D**) immunopositive staining for Vimentin (magnification ×40).

The EMT status was determined by analyzing both E-cadherin and Vimentin expression. Tumors expressing E-cadherin and/or lacking Vimentin were classified as epithelial, whereas tumors lacking E-cadherin and/or expressing Vimentin were classified as mesenchymal (Figure 2). When patients with epithelial and mesenchymal tumors were compared, regardless of the mucinous histology, patients with mesenchymal tumors were 1.6 years younger, and there were 9.4% more women among them (Table 2). No significant difference in the Mucin-1 score was observed between epithelial and mesenchymal tumors. Epithelial tumors were significantly better differentiated than mesenchymal tumors (*p* = 0.034). Patients with mesenchymal tumors had significantly more prominent tumor budding (*p* < 0.001).

Binomial and ordinal logistic regressions (adjusted for age and sex) were performed to evaluate the impact of Mucin-1, E-cadherin, and Vimentin on mucinous histology and disease severity. The results indicated Mucin-1 as an independent predictor of tumor differentiation (odds ratio of 3.312, 95% CI [1.418–7.737]; *p* = 0.006). Tumors with increased cytoplasmatic E-cadherin expression are 2.982-times more likely to have mucinous histology (95% CI [1.130–7.868]; *p* = 0.027), while the increase in cytoplasmatic the E-cadherin score was negatively associated with tumor grade (odds ratio of 0.228, 95% CI [0.072–0.722]; *p* = 0.012). Vimentin was identified as an independent predictor of tumor budding (odds ratio of 2.738, 95% CI [1.519–4.934]; *p* = 0.001).

**Figure 3:**
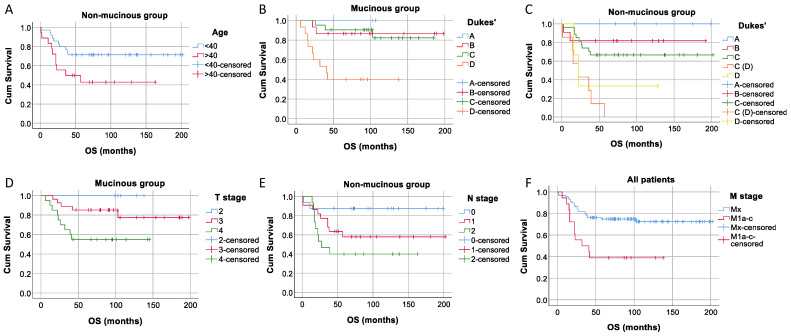
The Kaplan–Meier analysis of patients’ survival according to (**A**) the <40 and the >40 age groups in non-mucinous tumors; (**B**,**C**) the Dukes’ classification for mucinous and non-mucinous tumors, respectively; (**D**) the T stage for mucinous tumors, (**E**) the *N* stage for non-mucinous tumors, and (**F**) the Mx and M1a-c stages for all patients; all presented differences in overall survival are statistically significant (*p* < 0.05).

We tested if there was a significant difference in overall survival between patients with mucinous and non-mucinous tumors, epithelial and mesenchymal tumor types, as well as between positive and negative tumors for Mucin-1, membrane E-cadherin, cytoplasmatic E-cadherin, and Vimentin, and found no statistically significant differences in survival distributions for any of the investigated groups. We also performed a survival analysis for all collected patients’ demographics and clinical and pathological characteristics (as seen in Table 1) separately in the complete patients’ group and mucinous and non-mucinous groups. We found significant differences in survival time between the patients younger and older than 40 years of age in the non-mucinous group (*p* = 0.026), based on the Dukes’ classification for mucinous and non-mucinous groups (*p* = 0.001 for both), based on the T stage for mucinous tumors (*p* = 0.022), *N* stage for a non-mucinous group (*p* = 0.033), and between the Mx and M1a-c stages for all patients (*p* = 0.001) (Figure 3).

## 4. Discussion

The expression of EMT markers, E-cadherin, and Vimentin, along with the expression of Mucin-1 were analyzed in a cohort of patients with early-onset CRC in order to explore the role of EMT in mucinous histology. Up to date, the vast majority of studies dealing with EMT in cancer focused either on its role in metastatic disease, its potential as a prognostic factor in primary tumors, or its association with other relevant cellular processes, such as fibrosis [31]. In spite of its importance for clinical management, the potential significance of EMT in specific histological subtypes of malignant disease was neglected. Although mucins have been demonstrated to be involved in the EMT process and also in the enrichment of the cancer stem cell population in different cancer types [32], only a handful of recent studies investigated the EMT in mucinous histology [33,34,35].

This study was conducted in patients with early-onset disease, which represents a specific subset of CRC, characterized by a predominance of mucinous tumors [36]. This subset of patients was shown to harbor genetic and non-genetic determinants of risk for CRC, and these individuals would highly benefit from preventive measures [37,38].The total number of patients enrolled in this study is relatively small due to the age cutoff value and other inclusion criteria. However, the exclusion of patients older than 45 years, those with familial forms of cancer, ulcerative colitis or partial mucin production (below 50%), and those who underwent neoadjuvant chemoradiotherapy was expected to result in better patient stratification. Although our study was limited to tissue samples and did not characterize the underlying molecular mechanism of EMT in vitro, the expression of EMT markers in tumor tissue was studied in the invasive tumor front, since EMT occurs in this region when cancer cells come into contact with stromal cells, as well as various signaling molecules and stromal cell derivatives [39,40,41].

Considering the observed heterotopic expression of E-cadherin, we determined both the expression of membrane (MI) and cytoplasmic (CI) expression of E-cadherin. Patients with mucinous-type early-onset CRC had a statistically significantly higher E-cadherin CI, compared to patients with non-mucinous tumor histology. Total and MI scores for E-cadherin were also higher in mucinous compared to non-mucinous tumors but without statistical significance. Previous studies have shown that the aberrant expression of cytoplasmic E-cadherin may reflect an increased likelihood of metastasis [24]. Cytoplasmic E-cadherin expression in primary tumors was significantly higher in patients with recurrence during their follow-up, compared to those who did not have relapses, while it was lower in those who did not have primary metastases and in those who did not develop disease recurrences over time [24]. These findings suggest that cytoplasmic (aberrant) expression of E-cadherin reflects the unfavorable biological behavior of CRC [24]. Our study showed that in the group of mucinous CRC, a higher cytoplasmic (aberrant) expression of E-cadherin coincided with later tumor stage (28.2% vs. 5.7% stage IV), which also correlates with previous research [24].

The group of non-mucinous early-onset CRC patients had more Vimentin-positive patients and a higher vimentin score than the group with mucinous histology. Many studies showed that as cancer progresses, Vimentin expression increases, while E-cadherin expression decreases [13,42,43,44]. Colon epithelial cells normally show strong membrane expression of E-cadherin, which reflects the normal localization of this intercellular adhesive molecule [24]. When the expression of epithelial markers decreases and the expression of mesenchymal markers increases, the cells tend to separate from their place of origin. The process of EMT is associated with primary tumor growth, regional lymph node infiltration, vascular invasion, cancer grade and stage progression, tumor invasiveness, cancer progression to metastatic stage, and overall poor prognosis [13,45,46]. Although our study did not show a statistically significant difference in the overall survival in patients with increased cytoplasmic expression of E-cadherin, previous research showed an association between this aberrant expression in the invasive margin and adverse overall survival time in CRC [24,47]. Similarly, the Vimentin score was not significantly associated with overall survival in the present study, while previous research showed that an elevated expression of Vimentin can serve as a novel biomarker for worse prognosis and poor overall survival in CRC [16].

As expected, Mucin-1 expression was statistically higher in mucinous compared to non-mucinous tumors. Increased Mucin-1 expression was previously found to be a predictor of poor prognosis and overall survival in CRC, as it correlated with higher TNM stage, depth of invasion, lymph node, and distant metastases [18,48]. These findings suggest that Mucin-1 expression is a promising prognostic factor for CRC and may serve as a valuable biomarker for identifying the metastatic potential of the disease, but it may also be a promising target for future immunotherapy with the idea of reducing the risk of metastasis and increasing survival in patients with CRC [49,50].

Analysis of clinical and pathological data in our study indicates that early-onset disease, regardless of histology, is overrepresented in men, which correlates with other studies [10,51,52]. As expected, non-mucinous tumors had a lower grade than mucinous (94.3% vs. 73.6%) [53,54]. In the mucinous tumor group, there were more tumors of the right colon, while non-mucinous tumors were predominantly located in the left colon and rectum, as confirmed by other studies [8,55,56]. Additionally, hereditary mucinous forms are more frequently localized in the right colon in comparison to sporadic mucinous forms [57]. Lymphatic and vascular invasion was somewhat more pronounced in mesenchymal vs. epithelial tumors, and these tumors were of a higher Dukes stage at the time of diagnosis but without statistical significance.

Epithelial tumors were significantly better differentiated compared to mesenchymal (*p* = 0.034), which correlates with the finding that the preservation of E-cadherin correlates with better tumor differentiation [58]. Patients with mesenchymal tumors were 1.6 years younger, and there were 9.4% more women in this group in comparison to epithelial tumors. Patients with mesenchymal tumors had a statistically significantly more prominent tumor budding (*p* < 0.001), which correlates with recent studies that reveal the connection of tumor budding and EMT [59]. A loss or decreased expression of E-cadherin seen in tumor buds was also observed in our samples, which is consistent with previous reports [60,61]. Reduced expression or partial presence of E-cadherin on the membrane and heterotopic expression (alteration of membrane to cytoplasmic expression) was also observed in tumor buds, as in other studies [59,62]. 

## 5. Conclusions

Mucinous tumors had a significantly higher cytoplasmic E-cadherin score; they were significantly less differentiated, more advanced, and affected the right colon more frequently than other tumor locations in comparison to non-mucinous tumors. Epithelial tumors were significantly better differentiated and with less prominent tumor budding than mesenchymal tumors. The process of EMT appears to be more prominent in tumors in younger patients, regardless of mucinous histology. EMT status and especially cytoplasmic E-cadherin expression may represent useful tools for patient stratification and choice of therapy in early-onset CRC. This pilot study indicates some peculiarities in the status of EMT markers in younger CRC patients, and further studies are needed to reveal the underlying molecular mechanism of EMT in early-onset CRC.

## Figures and Tables

**Table 1:** Clinical and pathological characteristics of patients with non-mucionous and mucinous colorectal cancer.

	Non-Mucionous (*n* = 53)	Mucinous (*n* = 53)	*p* Value
Age, years	36.9 ± 5.2	35.7 ± 7.2	0.619
Male gender, %	66.0	66.0	1.000
Tumor localization, %			
rectum + left colon	81.1	60.4	0.039 *
right colon	18.9	39.6	
T stadium, %			
T2	13.2	11.3	0.587
T3	58.5	50.9	
T4	28.3	37.8	
*N* stadium, %			
N0	32.0	30.2	0.429
N1	30.2	41.5	
N2	37.8	28.3	
TNM stage, %			
I	9.4	3.8	0.027 *
IIA	13.2	20.8	
IIB	3.8	3.8	
IIC	3.8	3.8	
IIIA	3.8	0	
IIIB	43.4	28.3	
IIIC	16.9	11.3	
IVA	3.8	9.4	
IVB	1.9	0	
IVC	0	18.8	
Tumor grade, %			
G1 + G2	94.3	73.6	0.007 *
G3	5.7	26.4	
Tumor budding, %			
1	26.4	37.8	0.450
2	20.8	18.9	
3	52.8	43.3	
L positive, %	73.1	84.8	0.218
V positive, %	51.9	54.2	0.844
Mucin-1 score	2.07 ± 0.96	3.15 ± 0.64	<0.001 *
E-cadherin score			
total	1.84 ± 1.46	2.17 ± 1.09	0.112
membrane	0.84 ± 0.96	0.95 ± 0.89	0.206
cytoplasmatic	1.00 ± 0.62	1.21 ± 0.42	0.043 *
Vimentin score	0.85 ± 1.73	0.62 ± 1.47	0.473

* Statistically significant *p* value.

**Table 2:** Clinical and pathological characteristics of groups of colorectal cancer patients with epithelial and mesenchymal tumors.

	Epithelial (*n* = 51)	Mesenchymal (*n* = 55)	*p* Value
Age, years	37.1 ± 7.2	35.6 ± 6.0	0.143
Male gender, %	70.6	61.8	0.413
Tumor localization, %			
rectum + left colon	72.5	65.5	0.726
right colon	27.5	34.5	
T stadium, %			
T2	15.7	9.1	0.231
T3	58.8	50.9	
T4	25.5	40.0	
*N* stadium, %			
N0	39.2	23.6	0.091
N1	37.3	34.5	
N2	23.5	41.9	
TNM stage, %			
I	9.8	3.6	0.198
IIA	21.6	12.7	
IIB	2.0	5.4	
IIC	5.8	1.9	
IIIA	2.0	1.9	
IIIB	37.2	34.5	
IIIC	7.8	20.0	
IVA	2.0	10.9	
IVB	0	1.9	
IVC	11.8	7.2	
Tumor grade, %			
G1 + G2	92.2	76.4	0.034 *
G3	7.8	23.6	
Tumor budding, %			
1	43.1	21.8	<0.001 *
2	31.4	9.1	
3	25.5	69.1	
L positive, %	73.9	82.7	0.331
V positive, %	44.7	60.4	0.160
Mucin-1 score	2.56 ± 0.98	2.64 ± 0.98	0.716

* Statistically significant *p* value.

## Data Availability

Data supporting reported results can be obtained from the corresponding author upon request.
